# Density‐dependent sex‐biased development of macroptery in a water strider

**DOI:** 10.1002/ece3.6644

**Published:** 2020-08-05

**Authors:** Chang S. Han

**Affiliations:** ^1^ Department of Biology Kyung Hee University Seoul Korea

**Keywords:** density dependence, gene‐by‐environment, gene‐by‐sex, sex‐biased dispersal, *Tenagogerris euphrosyne*, wing dimorphism

## Abstract

In wing‐polymorphic insects, wing morphs differ not only in dispersal capability but also in life history traits because of trade‐offs between flight capability and reproduction. When the fitness benefits and costs of producing wings differ between males and females, sex‐specific trade‐offs can result in sex differences in the frequency of long‐winged individuals. Furthermore, the social environment during development affects sex differences in wing development, but few empirical tests of this phenomenon have been performed to date. Here, I used the wing‐dimorphic water strider *Tenagogerris euphrosyne* to test how rearing density and sex ratio affect the sex‐specific development of long‐winged dispersing morphs (i.e., sex‐specific macroptery). I also used a full‐sib, split‐family breeding design to assess genetic effects on density‐dependent, sex‐specific macroptery. I reared water strider nymphs at either high or low densities and measured their wing development. I found that long‐winged morphs developed more frequently in males than in females when individuals were reared in a high‐density environment. However, the frequency of long‐winged morphs was not biased according to sex when individuals were reared in a low‐density environment. In addition, full‐sib males and females showed similar macroptery incidence rates at low nymphal density, whereas the macroptery incidence rates differed between full‐sib males and females at high nymphal density. Thus complex gene‐by‐environment‐by‐sex interactions may explain the density‐specific levels of sex bias in macroptery, although this interpretation should be treated with some caution. Overall, my study provides empirical evidence for density‐specific, sex‐biased wing development. My findings suggest that social factors as well as abiotic factors can be important in determining sex‐biased wing development in insects.

## Graphical abstract

Long‐winged morphs developed more frequently in males than in females when individuals were reared in a high‐density environment. However, the frequency of long‐winged morphs was not sex‐biased when individuals were reared in a low‐density environment.

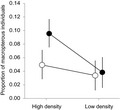

## 1 INTRODUCTION

Animals occasionally move from their natal habitat to another habitat to increase mating opportunities and to avoid inbreeding, competition or resource depletion (reviewed in Bonte et al., 2012). That is, animals disperse when the fitness benefit of dispersal exceeds the energetic costs of dispersal or increased predation risk during dispersal. In particular, in insects where wing polymorphism occurs, wing morphs greatly differ in their ability to disperse and possess advantages and disadvantages associated with their respective flight capability (reviewed in Guerra, 2011; Harrison, 1980; Roff, 1986a; Roff & Fairbairn, 1991; Zera & Denno, 1997). Long‐winged (macropterous) individuals can use their functional wings to escape from an unfavorable habitat and fly to another, distant habitat suitable for survival and reproduction, whereas short‐winged (micropterous or brachypterous) or wingless (apterous) individuals are flightless and sedentary. The differences in flight capability between wing morphs are further related to differences in other life history traits because of trade‐offs between flight capability and reproduction (reviewed in Guerra, 2011; Zera & Denno, 1997). Macropterous individuals tend to spend more energy developing wings and maintaining flight musculature but delay reproductive maturity. In contrast, micropterous or apterous individuals generally invest more energy in reproduction at the expense of flight‐related structures.

The frequency of macropterous individuals in wing‐polymorphic insect species can differ between males and females when the sexes face different costs and benefits of possessing wings (reviewed in Roff, 1990b; Zera & Denno, 1997). In insects, male‐biased macroptery is generally expected because the energetic costs of developing and maintaining flight muscles are higher in females carrying eggs than in males (Marden, 2000). In addition, when male–male scramble competition to increase mating frequency is strong, macroptery frequency is expected to be higher in males than in females. However, in insect species where females actively move to find limited egg‐laying substrates and compete to occupy them, macroptery frequency is expected to be higher in females than in males.

Given that sex bias in the intensity of intrasexual competition is one driver of differences in macroptery between males and females, the level of sex difference in macroptery is expected to depend on social environmental conditions such as the population density or the sex ratio. Population density during development is a cue indicating the level of mate or resource competition that will occur among conspecifics later in life (Kokko & Rankin, 2006). For example, when high rearing density increases the intensity of competition for mates or resources to a greater extent in males than in females, the advantage of flight capability is greater for males. This advantage can offset the costs associated with flight capability to a greater extent in males than it does in females. In such a scenario, it is possible that the development of long‐winged morphs is biased toward males. In addition to the effect of rearing density, the number of individuals of the opposite sex during development could be another important social factor determining wing development. As the encounter frequency between same‐sex and different‐sex individuals differs among sex ratio conditions, the levels of competition for mates will not be similar for a male living with many competitor males and one living with many females, even when they are in environments with the same density. Thus, the frequency of macropterous individuals is expected to be higher in males than in females under male‐biased sex ratio conditions, and vice versa under female‐biased sex ratio conditions.

Furthermore, when macroptery is heritable, environment‐dependent, sex‐biased macroptery can result from complex gene‐by‐environment‐by‐sex (G × E × S) interactions. First, sex differences in additive genetic variance or a weak cross‐sex genetic correlation indicate different gene expression between males and females, which is likely to enable the evolution of sexual dimorphism (Day & Bonduriansky, 2004; Ellegren & Parsch, 2007; Poissant, Wilson, & Coltman, 2010; Rhen, 2000). Thus, sex‐biased macroptery might also arise due to a sex‐specific genetic architecture for macroptery. In addition, as environmental conditions can alter the strength of cross‐sex genetic correlations (Berger et al., 2014; Fox, Czesak, & Wallin, 2004; Han & Dingemanse, 2017; Leips & Mackay, 2000; Punzalan, Delcourt, & Rundle, 2014; Simons & Roff, 1996; Vieira et al., 2000), social environmental conditions are expected to determine the level of the genetic contribution to sex‐specific macroptery. Despite empirical findings on sex differences in macroptery (reviewed in Roff, 1990b; Zera & Denno, 1997), the social environmental effects on sex‐specific development of long‐winged morphs and the underlying genetic basis have received little study.

**FIGURE 1:**
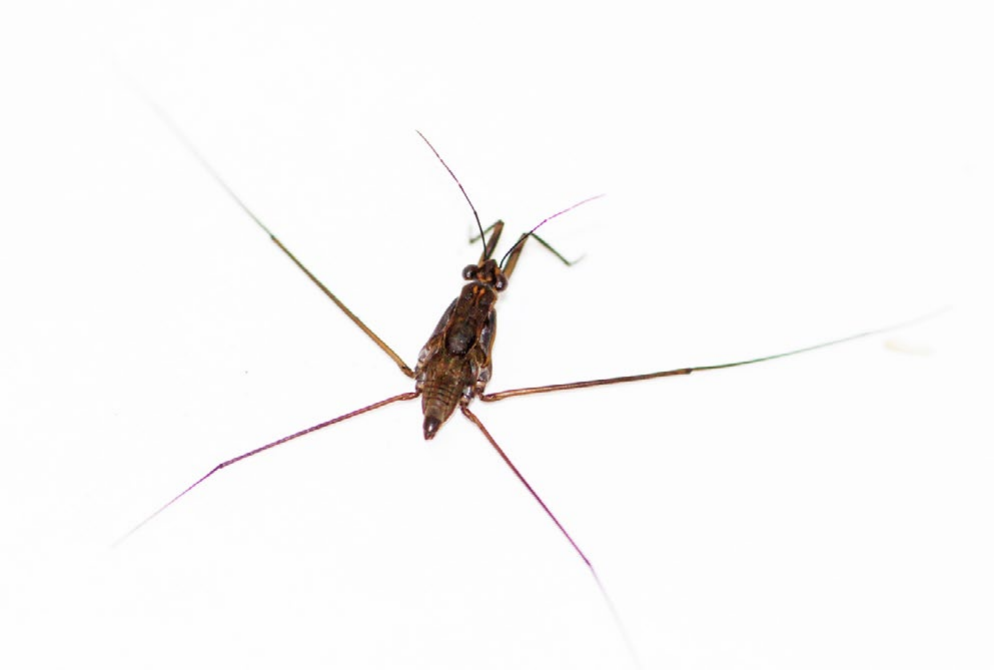
Adult male (apterous morph) of a water strider *Tenagogerris euphrosyne*. Photograph by Chang S. Han

In this study, I tested the effects of rearing density and sex ratio on the sex‐biased development of macropterous morphs and their genetic basis using a wing‐dimorphic Australian water strider, *Tenagogerris euphrosyne* (Heteroptera: Gerridae; Figure 1). Water striders are semi‐aquatic insects that inhabit a variety of temporary (e.g., pools, small streams and puddles) and permanent (e.g., lakes, oceans and rivers) water bodies (Spence & Andersen, 1994). *Tenagogerris euphrosyne* lives in relatively permanent habitats, such as large streams or ponds, and two distinct wing morphs occur: An apterous morph is common, but a macropterous morph occurs at a low frequency (5%–10%, Han & Brooks, 2013b). Macropterous morphs with a functional flight apparatus can leave unfavorable habitats and find favorable ones, whereas apterous morphs can move along the water surface but are not able to fly to other puddles or pools unconnected by riffles (Andersen, 1982, 1993, 2000; Fairbairn & Butler, 1990; Vepsäläinen, 1978). In an experiment, using virgin offspring from wild‐caught parents, I nested a density manipulation within a full‐sibling breeding design. I reared *T. euphrosyne* nymphs under two density regimes (low versus high) and measured the incidence of macroptery. First, I predicted a male‐biased incidence of macroptery in *T. euphrosyne* because of the sex‐specific trade‐off between flight capability and reproduction (reviewed in Guerra, 2011; Roff, 1990b; Zera & Denno, 1997). Female water striders, which are generally heavier than males, are expected to require more energy for flights (Marden, 2000), and intrasexual competition for mates is stronger in males than in females (Arnqvist, 1997).

In addition, if social environmental conditions such as density or the sex ratio strongly affect sex differences in terms of the fitness benefits and costs associated with wing development and maintenance, sex‐biased development of macropterous morphs is also expected in water striders. When both male–male competition and female costs associated with resistance to male harassment strongly increase under high density or a male‐biased sex ratio, those conditions can serve as cues to indicate the costs that an apterous morph will experience in the future habitat. Thus, a higher incidence of macroptery at a higher density or under a male‐biased sex ratio would be expected in both sexes. Previous research on *T. euphrosyne* has shown that sexual selection fluctuates across sex ratio conditions (Han & Brooks, 2013a, 2013b) and that rearing density affects mating behavior (Han & Brooks, 2015), implying a role of social environmental factors such as rearing density and sex ratio in determining the level of male–male competition and the female costs associated with resistance. However, the precopulatory mating harassment of females by males can depend on how long the pair bond will last. In the case of *T. euphrosyne*, the postmating guarding duration of males was much longer than that observed in other gerrids (Han & Brooks, 2013b). As a result, most females are occupied by males during the reproductive period, such that male harassment rates are not expected to increase at high densities or male‐biased sex ratios. Thus, I predicted that male–male competition for mates would be stronger at higher densities or male‐biased sex ratios, but that female costs associated with resistance do not increase at high densities or male‐biased sex ratios. This may lead to strong density and sex ratio‐dependent sex bias in macroptery in *T. euphrosyne*. Furthermore, as wing development is heritable in water striders (Fairbairn & King, 2009; Spence, 1989; Zera, Innes, & Saks, 1983), G × E, G × S, or the more complex G × E × S interaction may explain the environment‐dependent sex‐specific incidence of macroptery.

## 2 METHODS

### 2.1 Study species and rearing conditions

*Tenagogerris euphrosyne* is the most common water strider species in eastern Australia (Andersen & Weir, 2004) and is found in many freshwater habitats, such as streams, lakes, creeks, and ponds. I maintained laboratory stock populations of *T. euphrosyne* derived from offspring that were collected from a population in Piebald Creek, Atherton (Queensland, Australia; 17°16′S 145°28′E). In the laboratory, all of the water strider nymphs and adults were housed in the same room under a 14 hr:10 hr light/dark cycle at 28 ± 2°C which was similar to the natural environmental conditions during summer in Atherton. At the adult stage, the laboratory stock population was raised as 15–17 groups of 30 individuals (15 males and 15 females), and each group was kept in a large container (40 × 50 cm; water depth 10 cm). All the individuals in the stock population were mixed and re‐distributed into 15 groups every 4 days to ensure that the individuals were exposed to different competitors and mating partners. Multiple pieces of Styrofoam (2 × 30 cm; thickness 1–2 mm) were provided as resting sites. Frozen crickets (*Gryllus bimaculatus*) were provided as food every 2 days.

### 2.2 Experiment—Effects of rearing density and sex on wing development

**FIGURE 2:**
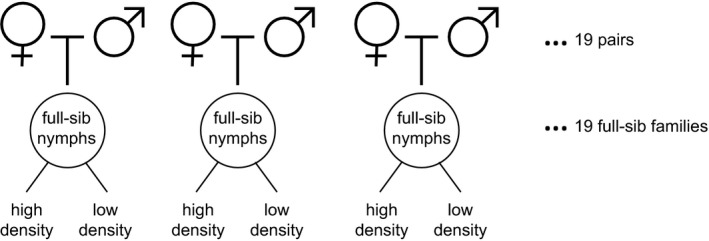
A full‐sib split‐brood breeding design. Nymphs of each full‐sib family were divided between low‐ and high‐density treatments. When they developed into adults, their sex and wing development were identified

For my experiment, I used the third generation of the stock population. When individuals in the laboratory stock population were close to adulthood, they were individually reared in containers (14 × 21 cm; water depth of 3 cm) to prevent mating. Next, I selected only apterous adults and implemented a full‐sibling split‐brood breeding design to test the effects of rearing density on the level of sex differences in macroptery. Each of the 19 unrelated males was singly mated with one female, producing 19 full‐sibling families (Figure 1). Upon hatching, full‐sibling 1st or 2nd instars were assigned to one of the following two density treatments: a relatively high‐density treatment (“high,” 20–25 full‐sibling nymphs in a container) and a relatively low‐density treatment (“low,” 4–6 full‐sibling nymphs in a container) (Figure 2). Nymphs in both density treatments were reared in containers of the same size (14 × 21 cm; water depth 3 cm), and individuals within each container were full‐siblings. I maintained up to 16 low‐density and 2 high‐density containers per full‐sib family. When the nymphs developed into adults, I distinguished the sexes of the individuals by genitalia morphology and noted whether each one had wings (macropterous or apterous). I also measured the sex ratios in each high‐density container. In total, I examined the wing development of 203 males reared at high nymphal density, 208 females reared at high nymphal density, 259 males reared at low nymphal density, and 215 females reared at low nymphal density.

### 2.3 Statistical methods

First, I used a generalized linear mixed‐effects model with a binomial error distribution and a logit link to test density‐dependent, sex‐biased wing development. I constructed a model in which the proportion of apterous individuals in each full‐sib container was fitted as the response variable, with full‐sibling family identity as a random effect and rearing density (two‐level factor: low vs. high), sex (two‐level factor: female vs. male), and their interaction as fixed effects. The residual variance was fixed at π^2^/3 according to standard procedures, and the estimates are on the latent scale (Nakagawa & Schielzeth, 2010).

In addition, to test the effect of sex ratio on wing development, I selected data from high‐density treatments and fitted another generalized linear mixed‐effects model where the sex ratio (the proportion of adult males in each full‐sib container), sex (two‐level factor: female vs. male), and their interaction were added as fixed effects, and full‐sibling family identity was added as a random effect. Because I reared only 4 nymphs per container in the low‐density treatments, only a few nymphs reached adulthood in each low‐density container and their sex ratios were easily biased by the sex of one or two individuals. Thus, I tested the effect of the sex ratio on wing development using only the high‐density treatment.

Finally, based on full‐sib families (*n* = 17) having offspring of both sexes in both density treatments, I calculated treatment‐specific correlations between the mean values for the incidence of macroptery (1 = macropterous, 0 = apterous) in males and females in each full‐sibling family to test the genetic basis of density‐dependent sex‐biased macroptery. As additive genetic correlations can be estimated simply as sire‐mean correlations (e.g., (Brooks, 2000; Hine, Lachish, Higgie, & Blows, 2002); reviewed in Astles, Moore, & Preziosi, 2006), I calculated cross‐sex correlations from the full‐sib family mean and used them as proxies for additive genetic correlations. I also statistically compared the direction of the treatment‐specific Pearson correlations via Fisher's *r* to *z* transformation (Diedenhofen & Musch, 2015). However, this comparison based on results from full‐sub families is not a robust test for G × E × S due to the limitations of my experimental design to calculate the additive genetic variance. Thus, this calculation provided the possibility that different G × S interactions may exist between different density treatments. All of the models were fitted using the “*glmer*” function (lme4 package) in R 3.0.2., and the “*sim*” function (arm package) was used to calculate the posterior distribution of the parameters based on 5,000 simulations (Gelman & Su, 2015). Significant differences were assessed using 95% confidence intervals.

## 3 RESULTS

**FIGURE 3:**
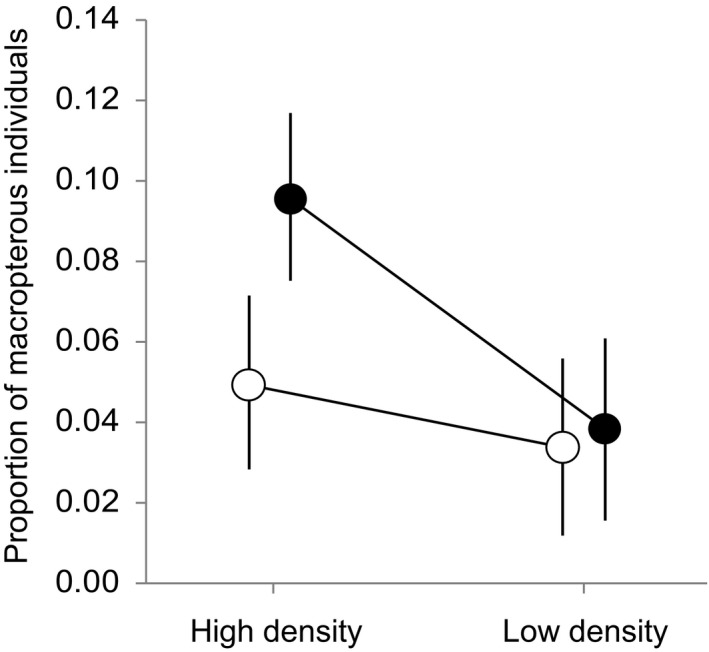
Rearing density‐specific development of macropterous males (filled circles) and females (open circles). The mean proportions of macropterous individuals with associated standard errors (error bars) were calculated using within‐family mean proportion values

Males showed a higher incidence of macroptery than females when they were reared under high‐density conditions (*β* = 1.18 [95% CI: 0.30, 2.05]), but no sex difference was noted in the incidence of macroptery when the individuals were reared under the low‐density treatment (*β* = −0.07 [95% CI: −1.07, 0.93]) (density × sex effect, Table 1, Figure 3). In the high‐density treatment, the sex ratio (M:F) varied from 0.18 to 0.75 but did not affect the incidence of macroptery in males or females (sex ratio × sex effect: *β*(*SE*) = −1.65(4.32), *z* = −0.38, *p* = .70; sex ratio effect: *β*(*SE*) = 3.47(3.60), *z* = −0.96, *p* = .34)). In addition, correlations between the proportions of macropterous individuals in males and females for each full‐sib family significantly differed between the density treatments (Fisher's *z* = 2.00, *p* = .04). The correlation was strongly positive in the low‐density treatment (*r* = 0.69, *n* = 17, *p* = .002) but was not different from zero in the high‐density treatment (*r* = 0.09, *n* = 17, *p* = .73).

**TABLE 1:** Effects of sex, rearing density, and their interaction on the incidence of macroptery

Fixed effects	*β* (95% CI)
Intercept	−3.49 (−4.31, −2.62)
Density^a^	0.05 (−0.97, 1.09)
Sex^b^	1.15 (0.30, 2.00)
Density × Sex^c^	−1.33 (−2.64, −0.09)

Random effects	Variance (95% CI)
Full‐sibling family ID	0.70 (0.35, 1.09)
Residual	π^2^/3

Note

Estimates are provided with 95% confidence intervals in parentheses.

^a^ Reference category was the high‐density treatment.

^b^ Reference category was the female sex.

^c^ Estimate indicates the effect of density on sex differences in the incidence of macroptery.

## 4 DISCUSSION

When water striders *T. euphrosyne* were reared at high density, the incidence of macroptery was higher in males than in females. High nymphal density suggests high levels of competition for resources (e.g., mates and food) later in life. As a result, the benefits associated with wing development (e.g., increased access to resources and chances to mate in a newly colonized habitat) are expected to exceed the costs (e.g., energetic costs of maintaining the flight‐related apparatus) in a high‐density environment. However, females reared at high nymphal density are also likely to face the risk of strong resource competition in the future, but no risk of reduced mating opportunities is expected because the sex ratios of *T. euphrosyne* are male‐biased in nature (Han & Brooks, 2013b). Indeed, the energetic costs associated with possessing a functional flight apparatus are likely to be greater for females than for males in water striders because females are heavier than males (Andersen, 1994; Fairbairn, 1990); therefore, more energy is needed to construct the wings and flight muscles (Marden, 2000). Therefore, even when population density is high, *T. euphrosyne* females living in relatively permanent habitats do not tend to develop wings but shift resources that would be used for the maintenance of the flight apparatus to egg production (reviewed in Guerra, 2011; Zera & Denno, 1997). This explains why the development of macropterous forms under high nymphal density is less favored in females than in males.

When water striders *T. euphrosyne* were reared at low density, there was no sex difference in the incidence of macroptery. Males develop fewer macropterous morphs in low‐density rearing environments where intraspecific competition over resources or mates is expected to be low in the future than in more competitive and higher density environments. In low‐density rearing environments, apterous males and females both are known to allocate to reproduction the resources that would otherwise be needed to maintain the flight apparatus (reviewed in Guerra, 2011; Zera & Denno, 1997); for example, apterous males of field crickets become reproductively mature earlier, increase their mating activity, and develop large weaponry (Crnokrak & Roff, 1995, 1998, 2000, 2002; Guerra & Pollack, 2007; Zeng & Zhu, 2012; Zhao, Chai, & Zhu, 2017). Apterous females also rapidly develop ovaries, increase their ovary mass, and elevate fecundity (i.e., flight‐oogenesis syndrome, reviewed in Guerra, 2011; Zera & Denno, 1997). Thus, both males and females of *T. euphrosyne* focus on reproduction rather than dispersal in low‐density environments and show a low incidence of macropterous morphs, which eliminates sex differences in the incidence of macroptery.

In contrast to my results, density‐dependent sex‐biased wing development was not observed in other water strider species (Harada & Spence, 2000; Harada, Tabuchi, & Koura, 1997). Harada et al. (1997) showed that the effects of nymphal density on the development of macroptery were similar between the two sexes in the water strider *Aquarius paludum*: A larger proportion of macropterous morphs emerged at high nymphal density than at low nymphal density in both males and females (Harada et al., 1997). This might be due to the species‐specific mating behavior of *A. paludum*. If *A. paludum* females experience a high degree of harassment by males as well as strong competition for food at high densities, they can also benefit by dispersing at high densities. In the case of *A. paludum*, where the duration of postmating guarding is shorter (e.g., less than 10 min) than in other gerrids (Arnqvist, 1997), females are usually unoccupied by guarding males and available for additional matings, such that single females are expected to experience a higher male harassment rate in a higher density environment, which incurs fitness costs (e.g., reduced foraging rates). In contrast, in the case of *T. euphrosyne,* for which the postmating guarding duration is much longer (an average of 5.4 days and a maximum of 13 days) than that of other water strider species (Han & Brooks, 2013b), most female water striders are guarded by males mounted on top of them regardless of density or sex ratio conditions. In addition, females in the mating position tend to be harassed less by single males than do females not in the mating position (C.S. Han, personal observation), which may increase their foraging rate. Consequently, the harassment rate by single males is expected to be similar between environments of different densities in *T. euphrosyne*. Overall, the benefits of dispersing at high densities are expected to be greater in *A. paludum* females than in *T. euphrosyne* females. Thus, in contrast to *T. euphrosyne*, *A. paludum* exhibits density‐dependent macroptery in both females and males. Therefore, I suggest that species‐specific life history, ecology, and mating patterns of males and females greatly influence environmental and sex differences in macroptery.

Contrary to my predictions, the incidence of macroptery was not affected by the sex ratio. This might be because *T. euphrosyne* nymphs at the critical period of wing development were not able to distinguish the sexes of other nymphs. However, as the sex of the nymphs that died before eclosion to adults was not recorded in my experiment, the lack of differences in macroptery among sex ratio conditions in *T. euphrosyne* might be due to missing data. In addition, although full‐sibling nymphs were reared with no adults in my experiment, *T. euphrosyne* nymphs generally grow in the presence of adults in natural environments. Thus, it is also possible that *T. euphrosyne* nymphs perceive the local sex ratio based on the sex of adults, rather than that of nymphs, during their development. Therefore, future studies are needed on the role of the sex ratio in the wing development of water striders to take such scenarios into account.

Furthermore, I showed that full‐sibling males and females showed a similar incidence of macroptery at low nymphal density, whereas full‐sibling males and females differed in the incidence of macroptery at high nymphal density. This finding implied a genetic basis for environment‐dependent sex‐biased macroptery, although this interpretation should be taken with some caution. This is because the variance attributable to full‐sibling families includes nonadditive genetic variances (e.g., dominance, epistatic, or maternal genetic variances) as well as additive ones and indicates an upper bound on the additive genetic variance (Falconer & Mackay, 1996; Lynch & Walsh, 1998). In order to overcome the limitation of using a full‐sib breeding design to estimate the genetic basis of traits, a robust breeding design such as a full‐sib/half‐sib breeding design should be used to calculate additive genetic variance and cross‐sex or cross‐environment genetic correlations. As a result, cross‐sex genetic correlations based on the full‐sib pedigree design in my experiment are expected to be biased by nonadditive genetic effects. The contribution of nonadditive genetic variances to macroptery might not be negligible. Although the magnitude of nonadditive genetic variances in water strider wing dimorphism has not been studied, wing development in the pea aphid *Acyrthosiphon pisum* was strongly influenced by maternal age (MacKay & Lamb, 1979), whereas the contribution of nonadditive genetic effects to wing dimorphism was found to be small in the field cricket *Gryllus firmus* (Roff, 1986b, 1990a). In my experiment, although it remains uncertain to what extent among‐family variance is biased by nonadditive effects, the moderate estimates of among‐family variance are likely to have additive effects. Thus, although my evidence is not conclusive, my work suggests that sex‐biased macroptery may arise via sex‐specific genetic architectures and that the environment during development may affect the expression of genetic variation in macroptery in a sex‐specific way, leading to environment‐dependent sex‐specific macroptery.

To the best part of my knowledge, the present study is the first evidence to show that the social environment during rearing affects sex‐specific macroptery. Moreover, although there is considerable evidence for environment‐dependent dispersal (reviewed in Matthysen, 2012) or sex‐biased dispersal (reviewed in Greenwood, 1980; Mabry, Shelley, Davis, Blumstein, & Van Vuren, 2013; Pusey, 1987; Trochet et al., 2016) in animals, the social environmental effects on sex‐specific dispersal are still poorly understood. For example, high‐density environments increase intraspecific resource competition in female tephritid flies *Paroxyna plantaginis*, which actively move to find suitable egg‐laying substrates, leading to increased female dispersal at high density (Albrectsen & Nachman, 2001). However, male tephritid flies tend to remain in their familiar territories and adopt a sit‐and‐wait mating strategy regardless of density environments (Albrectsen & Nachman, 2001). In addition, in the white‐tailed deer *Odocoileus virginianus*, young males generally leave their natal habitat, but females differ in dispersal distance according to the population density in their habitat (Lutz, Diefenbach, & Rosenberry, 2015). This is because the reproductive success of male white‐tailed deer is not affected by changes in home range or populational density, whereas female reproductive success decreases as the fawning habitat declines under high‐density conditions (Lutz et al., 2015). In the butterfly *Boloria eunomia*, an increase in the number of males in the population increased male sexual harassment, resulting in female dispersal, whereas a decrease in the number of females reduced male mating opportunities, resulting in male dispersal (Baguette, Vansteenwegen, Convi, & Nève, 1998). Given the key role of dispersal in population dynamics, with its effects on the genetic compositions and sex ratios of populations (Aars & Ims, 2000; Amarasekare, 2004; Ims & Andreassen, 2005), research on environment‐specific, sex‐biased dispersal can greatly contribute to forecasting how populations will respond to environmental changes.

In conclusion, my study provides empirical evidence for environment‐specific, sex‐biased macroptery in wing‐dimorphic insects. Furthermore, environmental conditions likely interact with the potential for evolutionary genetic constraints upon the evolution of sexual dimorphism in dispersal (Connallon & Clark, 2014; Connallon & Hall, 2016). Therefore, future research with rigorous breeding designs to study cross‐sex and cross‐environmental genetic correlations for dispersal or macroptery would strongly contribute to our understanding of the genetic basis of environment‐specific, sex‐specific dispersal in animals.

## CONFLICT OF INTEREST

None declared.

## AUTHOR CONTRIBUTIONS

**Chang S. Han:** Conceptualization (lead); formal analysis (lead); funding acquisition (lead); investigation (lead); methodology (lead); writing‐original draft (lead); writing‐review & editing (lead).

## Data Availability

All data are available at https://doi.org/10.5061/dryad.b5mkkwh9z.
